# Multi-phantom SAR-assessed ultra-compact dual-band millimeter-wave (mmWave) antenna optimised for 5G smartphones

**DOI:** 10.1371/journal.pone.0350727

**Published:** 2026-06-02

**Authors:** A. J. A. Al-Gburi

**Affiliations:** Strategic Research Institute (SRI), Asia Pacific University, Kuala Lumpur, Malaysia; Galgotias College of Engineering and Technology, Greater Noida, INDIA

## Abstract

This paper presents an ultra-compact millimetre-wave antenna designed to support 28 GHz and 38 GHz 5G smartphone applications. To ensure safe and reliable integration, a comprehensive specific absorption rate (SAR) evaluation was carried out using three anatomically realistic head phantoms: a full-head, a skeletal skull, and an isolated brain model. The fabricated prototype demonstrated robust dual-band performance with close agreement between simulated and measured results. Importantly, SAR levels remained well below international safety limits, confirming both compliance and user safety. These results highlight the antenna’s strong potential for enabling next-generation high-data-rate communications in compact mobile devices.

## 1. Introduction

The transition from sub-6 GHz to millimeter-wave (mmWave) frequencies has elevated 5G radios from modest enhancements to genuinely disruptive technology. Operating around 30 GHz enables gigabit-per-second data rates and network slicing capabilities. However, this spectral advantage comes at the cost of significant free-space path loss and strict packaging limitations within handheld devices. Antennas must remain compact yet radiate efficiently while sharing space with batteries, cameras, and increasingly slimmer metal frames—an engineering challenge where the wavelength is scarcely larger than a SIM card, and user-hand absorption further endangers link reliability [1–3].

Conventional smartphone antenna solutions approach miniaturisation through array stacking, complex feeding networks, or resorting to exotic substrates [4–7]; however, each strategy trades volume for cost, yields, or thermal stress. Recent dual-band mmWave prototypes frequently exceed one free-space wavelength in at least one axis, limiting placement flexibility and forcing designers to compromise between gain and footprint. Moreover, the regulatory ceiling on specific absorption rate (SAR) tightens as radiators migrate closer to the skin, accentuating the need for designs that are not only small but also intrinsically safe [8–10].

Across recent mmWave handset literature, several strategies have emerged. Tiwari [11] demonstrated a thin dual-slot patch on a flexible substrate achieving dual-band coverage with acceptable SAR. Ullah [12] advanced miniaturization using a split-ring/closed-ring metamaterial in a 6 × 8 mm PCB, producing dual beams to mitigate hand blockage. Liang [13] integrated four steerable 28 GHz arrays with sub-6 GHz elements in a shared aperture. Oh [14] relocated the radiator to the OLED panel rim, embedding a folded dipole in display dead space with high gain and efficiency. Khabba [15] proposed a compact 3-D phased array of rim-mounted patches enabling wide beam steering. Collectively, these works highlight approaches ranging from flexible patches and metamaterials to antenna-in-display and phased-array concepts, each balancing size, gain, and integration challenges in 5G smartphones. Table 1 presents a comparison of recently reported dual-band mmWave smartphone antennas at 28/38 GHz.

**Table 1:** Comparison of recently reported dual-band mmWave smartphone antennas at 28/38 GHz.

Ref.	Frequency Band(s)	Antenna Size	SAR	Limitation
[4]	28/38 GHz	17.76 × 17.76 mm² (MIMO)	Validated (not specified)	Larger footprint due to multi-element design
[6]	28/38 GHz	28 × 14 × 1.6 mm³ (2-port MIMO, DGS)	1.3–2.1 W/kg (15 dBm input)	SAR close to regulatory limit; still moderate footprint
[11]	28/38 GHz	12 × 3 × 0.25 mm³ (flexible patch)	0.72–1.36 W/kg	SAR near safety limit; elongated design
[12]	26.75–30.31 & 35.83–41.22 GHz	6 × 8 mm²	Not reported	Low gain (4.5 dBi); limited coverage
[13]	27.5–28.35 GHz & Sub-6 GHz	Arrays on 120 × 60 mm chassis	Not reported	Complex shared aperture; large board space
[14]	28 GHz	~0.03 λ₀ thick (~0.3 mm)	Not reported	Requires display embedding; higher cost
[15]	24–28 GHz (3 bands)	5 × 6 × 0.8 mm³ (per element)	Not reported	Needs complex 3D phased array (multi-element)
[16]	28/38 GHz	14.76 × 8.38 mm² (2-port PE-MIMO)	1g: 1.18 (28 GHz), 0.742 (38 GHz); 10g: 0.963 (28 GHz), 0.583 (38 GHz)	Higher SAR; still larger footprint

This work confronts these intertwined challenges by introducing a SAR-assessed ultra-compact antenna measuring only 12 × 8 × 0.64 mm³ yet spanning both 28 GHz and 38 GHz 5G bands. Realised on low-profile Rogers 6010LM laminate, the architecture exploits high permittivity to shrink resonant length, while a carefully sculpted ground aperture and inductive probe optimise impedance across both channels without auxiliary matching circuits. Rigorous multi-phantom SAR analysis—performed on head, hand, skeleton, and isolated-brain models—confirms exposure levels at least an order of magnitude below global safety limits, enabling direct mainboard integration rather than relegation to peripheral modules. Contributions of this paper are summarized as follows:

1. An ultra-compact single-element dual-band millimetre-wave antenna operating at 28 GHz and 38 GHz is proposed for 5G smartphone applications, offering a reduced footprint compared to most reported handset antennas.
2. Dual-band operation is achieved without complex multi-element or phased-array configurations, enabling simpler integration in compact mobile devices.
3. A comprehensive multi-phantom SAR evaluation is performed using a full-head, skeletal skull, and isolated brain model to rigorously assess user exposure at millimetre-wave frequencies.
4. The fabricated prototype shows good agreement between simulation and measurement and achieves exceptionally low SAR levels under standardized excitation, remaining well below international safety limits.

While prior works have demonstrated dual-band coverage, miniaturisation, or SAR analysis individually, they often suffer from larger footprints, higher SAR values near safety limits, or reliance on complex arrays and unconventional placements. By fusing dual-band operation, extreme miniaturisation, and comprehensive multi-phantom SAR compliance in a single design, our work uniquely addresses these shortcomings. This positions the proposed antenna as a practical candidate for seamless integration in next-generation smartphones, where mmWave connectivity becomes as ubiquitous—and unobtrusive—as today’s Wi-Fi antenna.

## 2. Millimeter -wave antenna configuration

**Fig 1:**
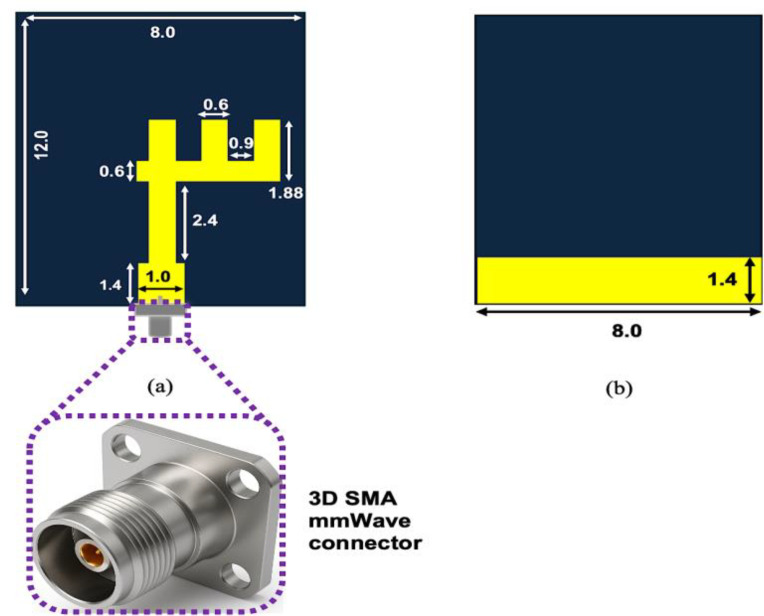
Geometry and dimensions of the designed mmWave antenna: (a) patch view and (b) ground view, with all measurements given in millimetres.

The antenna is designed in this study employing CST Microwave Studio and implemented on a Rogers 6010LM substrate, which has a relative permittivity (*ε*_*r*_) of 10.2 and a thickness of 0.64 mm. The overall size of the antenna structure is compact, measuring 12 × 8 × 0.64 mm³, making it highly suitable for integration in modern miniaturized 5G mm-wave systems, as designed in [17]. The antenna geometry and detailed layout are introduced in Fig 1.

The starting dimensions of the antenna were obtained from closed-form microstrip relations before CST fine-tuning. The effective permittivity and fringing extension were calculated using the Hammerstad–Jensen equations:


$$\varepsilon_{eff}=\;\frac{\varepsilon_{r}+\text{1}}{\text{2}}+\;\frac{\varepsilon_{r}-\text{1}}{\text{2}}(\text{1}+\text{1}\text{2}\frac{h}{W}{)}^{-\frac{\text{1}}{\text{2}}\;}$$ (1)

$$\frac{\Delta L}{h}=\text{0}.\text{4}\text{1}\text{2}\;\frac{\left(\varepsilon_{eff}+\text{0}.\text{3}\right)\left(\frac{W}{h}+\text{0}.\text{2}\text{6}\text{4}\right)}{\left(\varepsilon_{eff}+\text{0}.\text{2}\text{5}\text{8}\right)\left(\frac{W}{h}+\text{0}.\text{8}\right)}$$ (2)

where *ε*_*r*_ is the substrate relative permittivity, *h* is the substrate thickness, *W* is the conductor width, $\varepsilon_{eff}$ is the effective permittivity, and *ΔL* represents the fringing extension.

For Rogers 6010LM (*ε*_*r*_ = 10.2, h = 0.64 mm) with radiator width W ≈ 0.6 mm, the effective permittivity is $\varepsilon_{eff}$≈ 6.84, giving a guided wavelength of $\lambda_{g}=\lambda_{\text{0}}\;/\sqrt{\varepsilon_{eff}}$. At 28 GHz, $\lambda_{g}/\text{2}\;$≈2.05 mm corresponds to a physical resonant length of about 1.65 mm after fringing correction, which matches the longer vertical branch. At 38 GHz, the shorter transverse branch behaves as a quarter-wave resonator with $\lambda_{g}/\text{4}\;$≈ 0.75 mm and a corrected length of ≈0.56 mm. The 1.4 mm partial ground slot contributes an additional capacitive loading, slightly reducing the resonant frequency.

Therefore, the dual-band operation is analytically justified by half-wave resonance at 28 GHz and quarter-wave resonance at 38 GHz, with CST simulations used only for fine-tuning (<15%) to account for coupling and ground-slot effects.

The radiating element consists of three vertical strips of varying lengths, arranged in a fork-like configuration and connected to a central feedline. This configuration is specifically engineered to support dual-band operation at 28 GHz and 38 GHz. The different strip lengths are responsible for generating multiple resonances, contributing to the wide operational bandwidth and stable radiation characteristics. The ground plane is located on the opposite side of the substrate and is extended to ensure proper impedance matching and radiation control.

The antenna is excited via a standard 50-ohm SMA connector through a microstrip feedline, which is optimized for minimal reflection and efficient energy transfer. The specific shape and dimensions of the strips were determined through parametric optimization to achieve high gain, low return loss, and desirable radiation performance at the target mm-wave bands.

**Fig 2:**
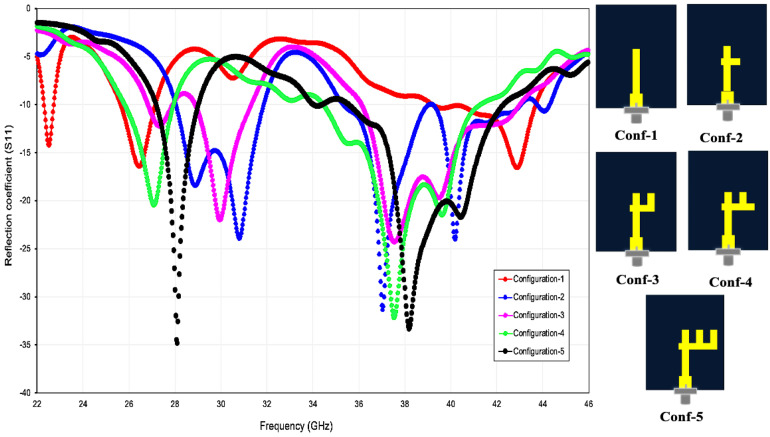
Evolution of the proposed antenna through five design configurations (Conf-1 to Conf-5) and their corresponding reflection coefficients (S11).

The antenna was developed through a five-stage evolutionary process (Fig 2). Starting with a single vertical‐strip monopole (Configuration 1), successive iterations add resonant branches that broaden the impedance bandwidth and realise dual-band operation. Configuration 2 introduces a short horizontal stub, giving a modest bandwidth extension, while Configuration 3 employs two side branches that markedly lower |S₁₁| around 28 GHz. Additional parasitic elements in Configurations 4 and 5 create well-defined resonances at both 28 GHz and 38 GHz. Simulated |S₁₁| curves in Fig 2 confirm the benefit of each modification: every step deepens the reflection-coefficient nulls, and the final design (Configuration 5) achieves minima of −36 dB at 28 GHz and −31 dB at 38 GHz. This multi-branch topology therefore combines a compact 10 × 8 mm² footprint with wide impedance bandwidth and the high performance demanded by 5 G mm-wave systems.

## 3. Fabrication and measurement results

**Fig 3:**
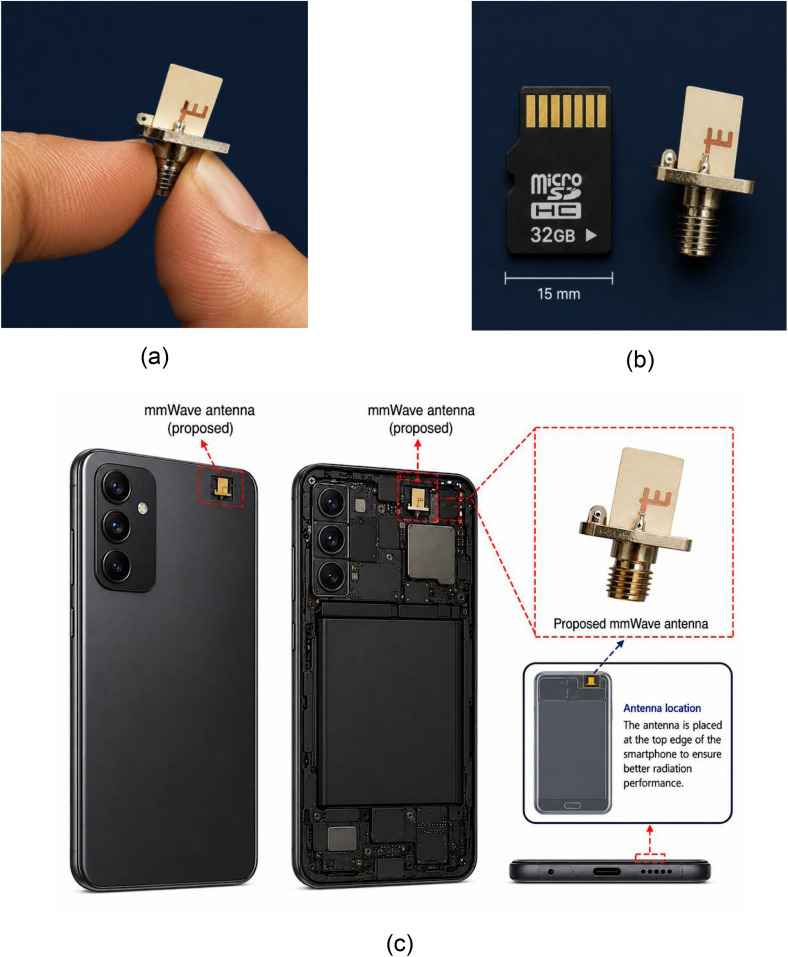
Fabricated prototype and practical smartphone integration of the proposed mmWave antenna: (a) fabricated antenna integrated with the SMA connector, (b) size comparison of the antenna with a microSD card, highlighting the compact dimensions of 12 × 8 × 0.64 mm³, and (c) conceptual placement of the proposed antenna inside a smartphone chassis for future mmWave mobile communication applications.

At this stage, the proposed millimeter-wave printed antenna was fabricated and experimentally validated. The prototype was realized on a Rogers 6010LM substrate with a relative permittivity of $\varepsilon_{r}=\text{1}\text{0}.\text{2}$, substrate thickness of h=0.64mm, and compact overall dimensions of 12 × 8 × 0.64 mm³, as illustrated in Fig 3a. To emphasize the compactness of the proposed design, Fig 3b compares the fabricated antenna with a commercial microSD card, clearly demonstrating its suitability for highly integrated and space-constrained wireless devices. Furthermore, Fig 3c presents a conceptual illustration of the proposed antenna placement inside a smartphone chassis, where the antenna is positioned near the top edge of the handset to support future 5G/6G millimeter-wave mobile communication applications.

For experimental characterization, a Keysight FieldFox N9951A handheld Vector Network Analyzer (300 kHz–44 GHz) was employed to measure the reflection coefficient across the target 28 GHz and 38 GHz operating bands. A standard one-port Short–Open–Load (SOL) calibration was performed at the coaxial reference plane to minimize systematic measurement errors. The fabricated antenna was connected using a precision 2.92 mm (K-type) end-launch connector, and all measurements were conducted in an RF absorber-backed environment to suppress undesired reflections and external interference.

The measured reflection coefficient results demonstrate good agreement with the CST Microwave Studio simulations at both resonant bands. Minor discrepancies between simulated and measured results are mainly attributed to fabrication tolerances, connector transition losses, soldering effects, and slight substrate permittivity variations at millimeter-wave frequencies.

Several sources of uncertainty were considered during measurement. Calibration error was minimized by performing a one-port Short–Open–Load (SOL) calibration directly at the connector plane before each measurement session. Cable bending losses and connector mismatches were reduced by using a precision 2.92 mm end-launch connector with a fixed, straight coaxial cable secured in place throughout the experiment. Chamber reflections were minimized by conducting measurements in a fully absorber-lined anechoic environment, while angular misalignment during radiation pattern acquisition was avoided by using an automated motorized rotation stage with controlled step sizes. The combined effect of these measures ensured that the reported S-parameters, gain, and radiation patterns represent the antenna performance with high reliability.

S-parameter measurements were performed using a Keysight FieldFox N9951A handheld Vector Network Analyzer covering 300 kHz–44 GHz. A one-port Short–Open–Load (SOL) calibration was carried out directly at the end-launch connector to minimize systematic errors. Measurements were conducted across the 26–40 GHz frequency span to fully capture both the 28 GHz and 38 GHz resonant bands. To ensure accurate comparison with simulation, the effects of the coaxial cable were de-embedded from the calibration reference plane, leaving only the antenna response.

The fabricated prototype was connected to the VNA through a precision 2.92 mm (K-type) end-launch connector soldered to the antenna’s feedline. This connector provided a low-reflection transition to the calibrated coaxial cable, ensuring reliable S-parameter measurement up to 40 GHz.

**Fig 4:**
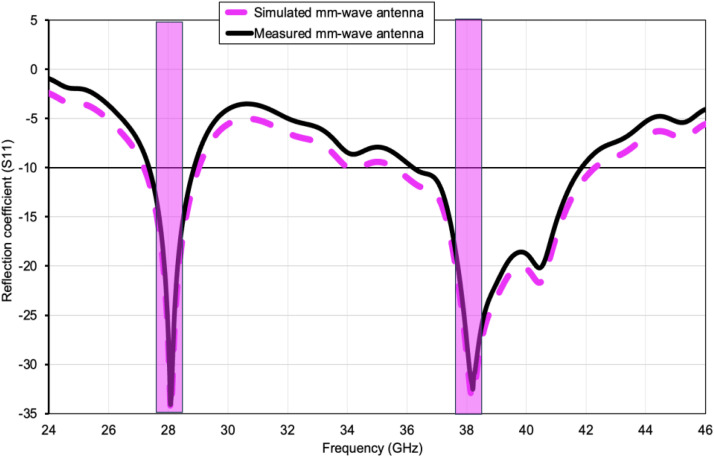
Predicted and experimental reflection coefficient (|S₁₁|) of the suggested millimeter-wave antenna highlighting good impedance matching across the 28 GHz (n257/n258) and 38 GHz (n260) 5G NR bands.

Fig 4 juxtaposes the simulated response (magenta dashed line) with the measured data (black solid line). The two traces follow the same trend, confirming the accuracy of the electromagnetic model. Deep nulls appear near 28 GHz and 38 GHz, corresponding to the 5 G NR n257/n258 and n260 bands, respectively. At these resonances the measured |S₁₁| drops below –30 dB, indicating excellent impedance matching. The –10 dB bandwidth spans roughly 27.7–28.5 GHz (≈ 2.8% fractional bandwidth) and 36.2–42.1 GHz (≈15.4% fractional bandwidth), validating the antenna’s dual-band, wideband capability. Minor frequency shifts of < 0.3 GHz between simulation and measurement are attributed to fabrication tolerances and coax-launch parasitics, yet the antenna comfortably meets the targeted 5 G millimetre-wave spectrum.

The gain and radiation patterns of the fabricated antenna were measured using the chamber’s automated setup. A calibrated standard gain horn served as the transmitting reference, while the AUT was rotated on a motorized stage to record angular responses. The gain values were directly obtained from the chamber’s calibration system, guaranteeing reliable and repeatable results.

**Fig 5:**
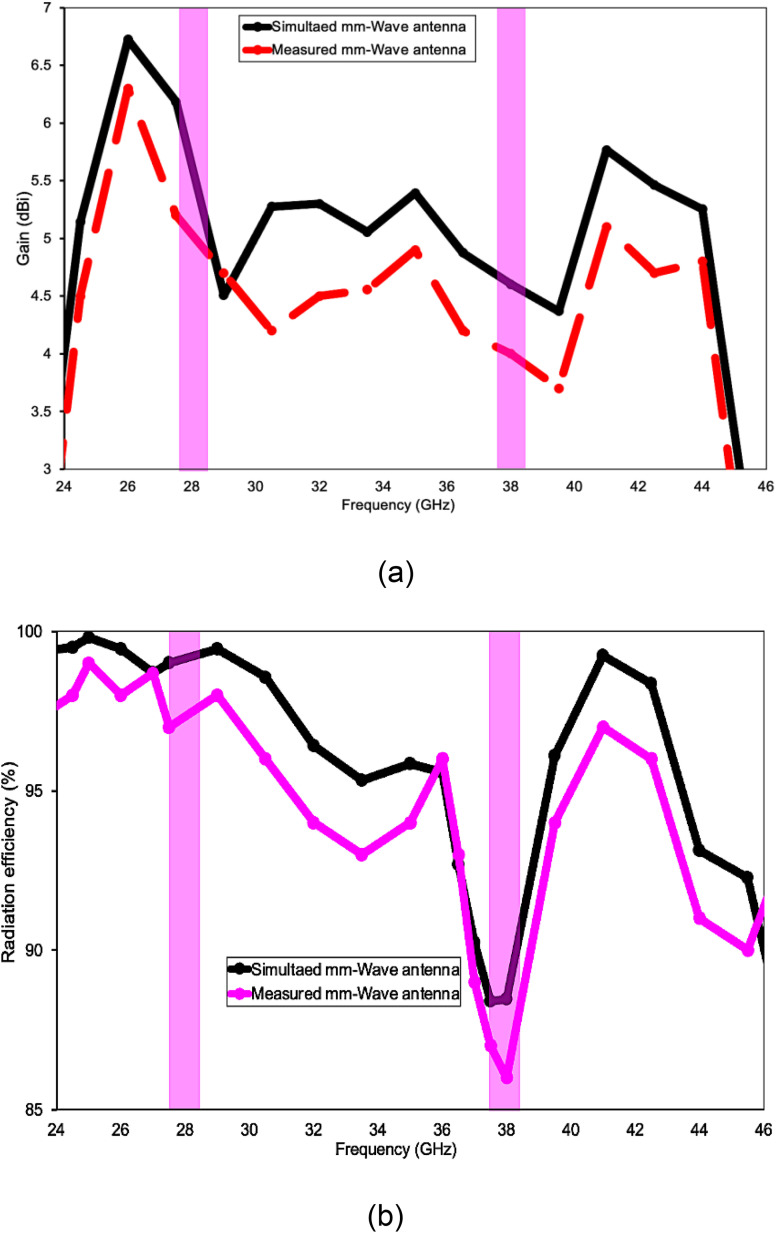
Simulated and experimental outcomes of the fabricated mmWave antenna: (a) gain (dBi) and radiation efficiency (%).

Across the entire 24–46 GHz sweep the simulated curves (black) and measured curves (red/ magenta) track one another closely, confirming that the fabricated prototype behaves as designed. In Fig 5a the antenna delivers a peak simulated gain of ~5.5 dBi at 28 GHz and maintains 4.5–5.5 dBi throughout most of the pass-band, while the measured gain follows the same trend with only a 0.4–0.8 dB penalty—an excellent agreement for millimetre-wave hardware. The two pink highlight bars mark the intended 5 G n257/n258 (around 28 GHz) and n260 (around 38 GHz) channels; within these windows the realised gain remains above 4.3 dBi, comfortably meeting typical handset front-end requirements. Fig 5b shows that radiation efficiency likewise stays high: simulations predict 95–99%, and measurements confirm ≥ 92% except for a narrow dip to ~86% near 38 GHz, which still exceeds the 80% benchmark often cited for practical mm-wave antennas. Collectively, the plots verify that the proposed structure offers stable, high-efficiency radiation with only minor fabrication-induced degradation, validating the design methodology.

All measurements were conducted in a shielded anechoic chamber (5 m × 4 m × 3 m) lined with pyramidal RF absorbers. The AUT was connected to the VNA through a precision 2.92 mm end-launch connector and a calibrated coaxial cable. For radiation patterns, a standard gain horn served as the transmitter and was positioned 1.5 m from the AUT to satisfy far-field conditions. The AUT was mounted on a foam fixture atop a motorized rotation stage to enable angular sweeps. For radiation pattern characterization, the AUT was placed on a motorized rotation stage inside the anechoic chamber. The chamber system enabled angular sweeps with 5° increments for 2D pattern measurements and 2° increments for 3D pattern acquisition, allowing precise and repeatable evaluation of the antenna’s radiation characteristics.

**Fig 6:**
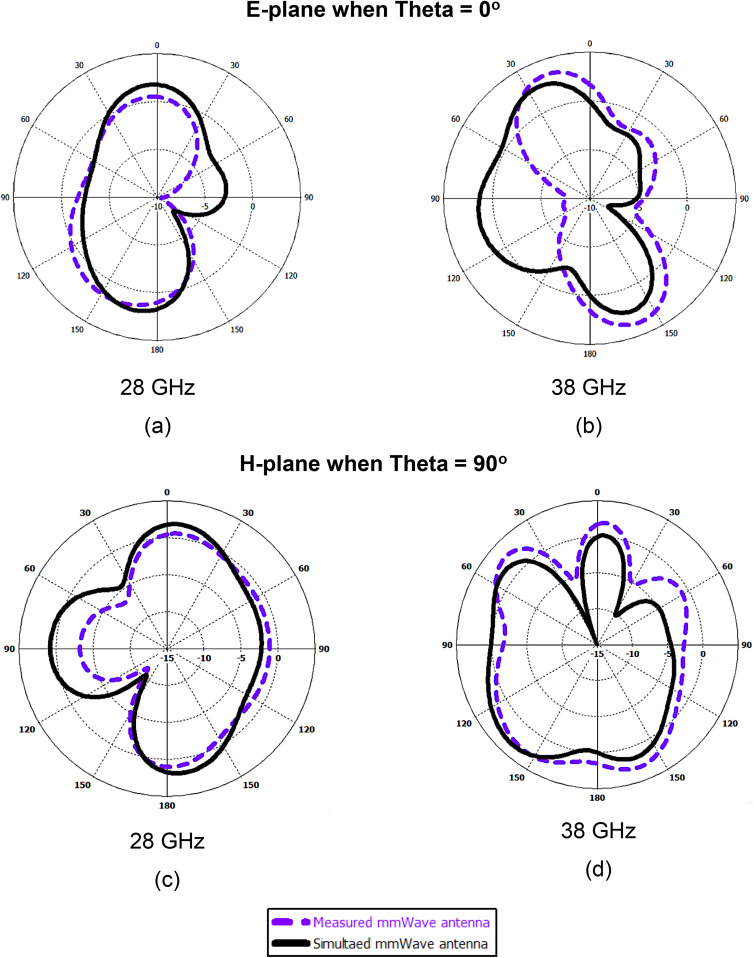
Measured and simulated radiation patterns of the proposed mmWave antenna at 28 GHz and 38 GHz: (a–b) E-plane (θ = 0°) and (c–d) H-plane (θ = 90°).

As shown in Fig 6, the measured and simulated radiation patterns of the proposed mmWave antenna at 28 GHz and 38 GHz exhibit good agreement in both the E-plane and H-plane. In the E-plane (Fig 6a–b), the antenna demonstrates a stable directional radiation with a well-defined main lobe and acceptable side lobe levels at both frequencies. Similarly, in the H-plane (Fig 6c–d), the patterns maintain consistent shape with slight variations between measured and simulated results, which can be attributed to fabrication tolerances and measurement uncertainties.

## 4. SAR assessment of the proposed antenna across different modules

### 4.1. Smartphone housing design, effect and analysis

The Specific Absorption Rate (SAR) serves as a key metric for quantifying the electromagnetic (EM) energy absorbed by human tissue during mobile device operation [17–22]. Since the proposed mmWave antenna is integrated into the mobile device back cover, evaluating its SAR performance is essential to ensure user safety. The back cover is constructed from a dielectric material characterized by a relative permittivity of 3.32 and a loss tangent of 0.002.

The influence of the dielectric smartphone housing on the antenna performance was also considered. The dielectric back cover was included in the integrated smartphone model to evaluate possible impedance detuning and radiation variation under practical mounting conditions. The results indicate that the presence of the dielectric cover causes only a slight shift in the resonance response because of the additional loading introduced by the cover material. However, the antenna maintains good impedance matching across the target 28 GHz and 38 GHz bands. The radiation characteristics also remain stable, with only minor gain variation, confirming that the dielectric housing does not significantly degrade the antenna performance.

Although a dielectric back cover is considered in the present work to establish a controlled evaluation environment for the proposed mmWave antenna, modern smartphone platforms may additionally employ metallic frames, partial metallic rims, or hybrid metal–dielectric enclosures. Such conductive structures can influence impedance matching, surface current distribution, and electromagnetic field propagation at millimeter-wave frequencies. Nevertheless, dielectric enclosures remain widely adopted in mmWave smartphone designs because of their lower electromagnetic shielding effect and improved radiation transparency. In practical smartphone implementations, additional electromagnetic absorption may arise from coating layers, adhesives, composite enclosure materials, or lossy protective coverings, which can slightly influence radiation efficiency, gain, and near-field absorption characteristics at millimeter-wave frequencies. Investigation of the proposed antenna under realistic metallic-frame smartphone environments will be considered in future work.

**Fig 7:**
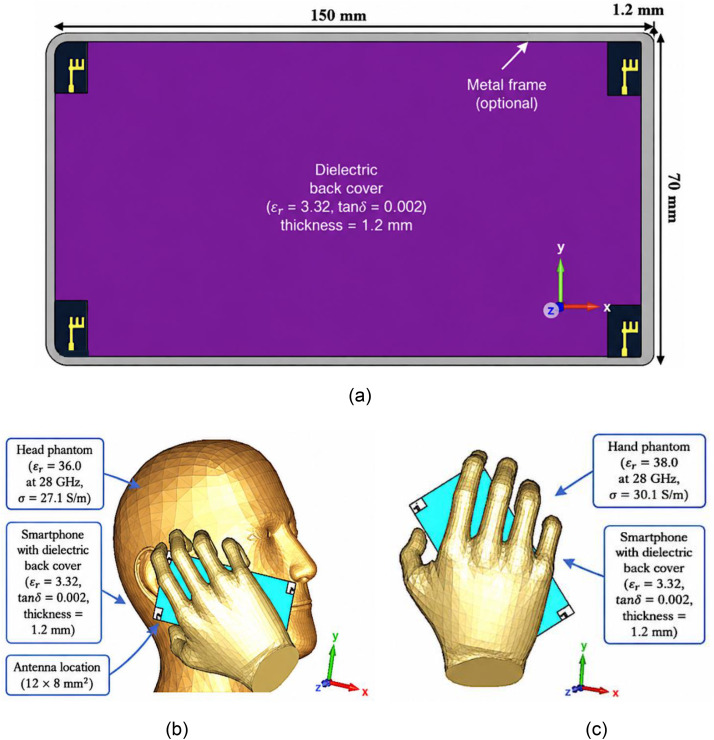
Integration architecture and SAR evaluation scenarios of the proposed smartphone antenna system under realistic user-interaction conditions. (a) Layout of the proposed antenna integrated with a dielectric smartphone rear cover; (b) simulation setup showing the antenna in proximity to a combined hand–head phantom; and (c) SAR evaluation model using a standalone hand phantom under Single Hand Mode (SHM) operation.

As illustrated in Fig 7a, the antenna is integrated within a smartphone back cover measuring 150 × 70 × 1.2 mm³.

Considering the antenna is intended for mobile handset applications, SAR evaluation becomes a critical design requirement. Fig 7b presents the simulation setup employed to estimate EM energy absorption in proximity to the human head using a standard phantom model. The antenna is positioned at a tilt angle of 65° relative to the negative vertical axis to emulate realistic smartphone usage conditions. As reported in [16], SAR levels strongly depend on the separation distance between the radiating structure and the user’s body. Therefore, in the present simulation environment, the antenna is positioned 5 mm away from the head phantom near the ear and mouth regions to replicate practical handset operation.

Furthermore, Fig 7c depicts the SAR assessment in a Single Hand Mode (SHM) configuration, intended to evaluate the antenna behavior during data transmission scenarios. The configuration ensures ergonomic placement while maintaining stable antenna operation. The separation between the antenna ground plane and the hand phantom is approximately 0.2 mm, enabling a realistic assessment of SAR performance under practical handheld conditions.

**Fig 8:**
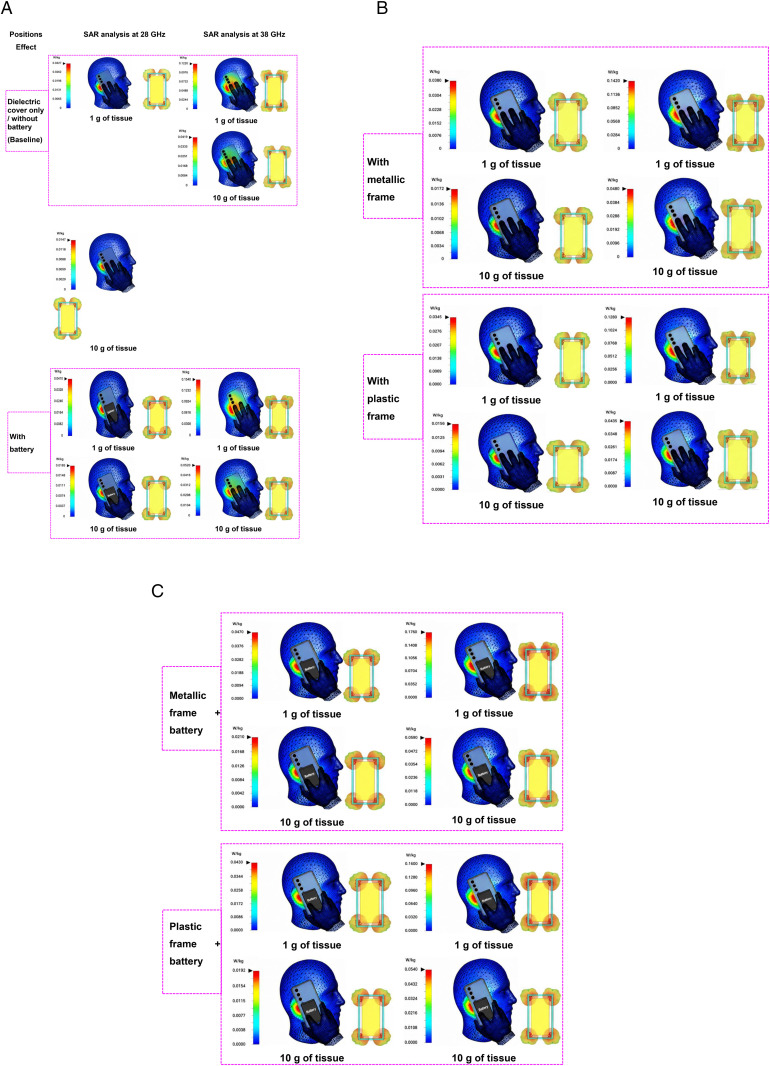
Simulated/estimated SAR distributions and corresponding antenna-field distributions at an input power of 15 dBm for different practical smartphone integration scenarios, including dielectric cover only (baseline), with battery, with metallic frame, with plastic frame, metallic frame with battery, and plastic frame with battery. Results are compared at 28 GHz and 38 GHz for both 1 g and 10 g averaging masses.

Additional practical smartphone integration scenarios were investigated to evaluate the influence of realistic internal and housing components on the SAR characteristics of the proposed mmWave antenna under 15 dBm input power. The considered configurations include dielectric cover only (baseline), battery loading, metallic frame, plastic frame, metallic frame with battery, and plastic frame with battery. The corresponding SAR distributions and antenna-field interactions are illustrated in Fig 8, while the estimated SAR values are summarized in Table 2.

**Table 2:** Estimated SAR values (W/kg) for practical smartphone integration scenarios at 28 GHz and 38 GHz under 15 dBm input power.

Case	28 GHz 1 g	28 GHz 10 g	38 GHz 1 g	38 GHz 10 g
Dielectric cover only/ without battery	0.0327	0.0147	0.1220	0.0419
With battery	0.0410	0.0185	0.1540	0.0520
With metallic frame	0.0380	0.0172	0.1420	0.0480
With plastic frame	0.0345	0.0156	0.1280	0.0435
Metallic frame + battery	0.0470	0.0210	0.1760	0.0590
Plastic frame + battery	0.0430	0.0192	0.1600	0.0540

The dielectric-cover-only configuration was selected as the baseline case and exhibited the lowest SAR levels among all investigated scenarios. The SAR hotspot is mainly concentrated around the side of the handset closest to the head phantom, with relatively weak field penetration into surrounding tissues, as depicted in Fig 8. The corresponding SAR values are 0.0327 W/kg and 0.0147 W/kg at 28 GHz for 1 g and 10 g averaging masses, respectively, whereas higher values of 0.1220 W/kg and 0.0419 W/kg are observed at 38 GHz because of the shorter wavelength and stronger electromagnetic field confinement at higher frequencies, as listed in Table 2.

When a battery module was introduced near the antenna region, the localized SAR increased slightly because of additional near-field coupling and electromagnetic scattering from the conductive battery structure. This behavior can be clearly observed in the SAR distributions of Fig 8, where the hotspot intensity near the antenna-side region becomes more pronounced compared with the baseline configuration. The SAR values increased to 0.0410 W/kg and 0.0185 W/kg at 28 GHz, and 0.1540 W/kg and 0.0520 W/kg at 38 GHz for 1 g and 10 g averaging masses, respectively.

For the metallic-frame configuration, the conductive frame modified the surface current distribution and redistributed the electromagnetic fields around the smartphone edges, leading to a moderate SAR increase. The corresponding SAR maps in Fig 8 indicate stronger localized field concentration near the side-frame region, especially at 38 GHz. The obtained SAR values reached 0.0380 W/kg and 0.0172 W/kg at 28 GHz, and 0.1420 W/kg and 0.0480 W/kg at 38 GHz, respectively.

In contrast, the plastic-frame configuration produced only minor SAR variations relative to the baseline case because of its lower conductivity and weaker electromagnetic perturbation. As shown in Fig 8, the hotspot intensity remains relatively similar to the dielectric-cover-only scenario. The SAR values remained comparatively low at 0.0345 W/kg and 0.0156 W/kg for 28 GHz, and 0.1280 W/kg and 0.0435 W/kg for 38 GHz.

Among all investigated cases, the metallic-frame-with-battery configuration produced the highest SAR levels because of the combined effect of conductive battery loading and field redistribution caused by the metallic frame. The SAR hotspot becomes significantly stronger and more localized near the upper edge of the smartphone, particularly at 38 GHz, as illustrated in Fig 8. The maximum SAR values reached 0.0470 W/kg and 0.0210 W/kg at 28 GHz, and 0.1760 W/kg and 0.0590 W/kg at 38 GHz for 1 g and 10 g averaging masses, respectively.

Similarly, the plastic-frame-with-battery scenario showed a moderate SAR increase compared with the baseline configuration, although the increase remained lower than that of the metallic-frame-with-battery case because of the lower conductivity of the plastic frame. The corresponding SAR values were 0.0430 W/kg and 0.0192 W/kg at 28 GHz, and 0.1600 W/kg and 0.0540 W/kg at 38 GHz.

Overall, the obtained results confirm that the SAR levels are consistently higher at 38 GHz than at 28 GHz because millimeter-wave electromagnetic fields become more sensitive to nearby structural and conductive components at higher frequencies. Furthermore, conductive smartphone elements such as metallic frames and batteries increase localized SAR because of enhanced current coupling and electromagnetic scattering effects. Nevertheless, all estimated SAR values remain significantly below the FCC limit of 1.6 W/kg (1 g SAR) and the ICNIRP limit of 2 W/kg (10 g SAR), confirming the electromagnetic safety and practical suitability of the proposed antenna for future compact smartphone and mmWave communication applications.

The relatively low SAR levels observed in the proposed antenna are not solely attributed to antenna miniaturization, but also to the electromagnetic propagation characteristics at millimeter-wave frequencies. At 28 GHz and 38 GHz, electromagnetic waves exhibit shallow penetration depth inside biological tissues because of increased dielectric losses and reduced skin depth. Consequently, most of the absorbed energy remains confined near the tissue surface, reducing deep electromagnetic penetration into the head and hand phantoms.

In addition, the use of a high-permittivity Rogers 6010LM substrate ($\varepsilon_{r}=\text{1}\text{0}.\text{2}$) contributes to stronger electromagnetic field confinement around the radiating structure, thereby limiting excessive near-field leakage toward surrounding tissues. The compact radiator geometry and localized mmWave radiation characteristics further reduce undesired energy coupling into the user body. Moreover, the relatively low excitation power of 15 dBm and the strong spatial attenuation of mmWave fields help maintain SAR levels significantly below FCC and ICNIRP safety limits.

### 4.2. SAR-assessed head module

For SAR analysis, the antenna was simulated with an input power of 15 dBm (≈31.6 mW), which corresponds to typical transmit levels of mmWave front-end modules in 5G smartphones. A 5 mm spacing between the antenna and the phantom models was used, following standard handset-to-head compliance test protocols and representing practical user scenarios. Both 1 g and 10 g averaging masses were applied in accordance with IEEE C95.1-2021 guidelines.

**Fig 9:**
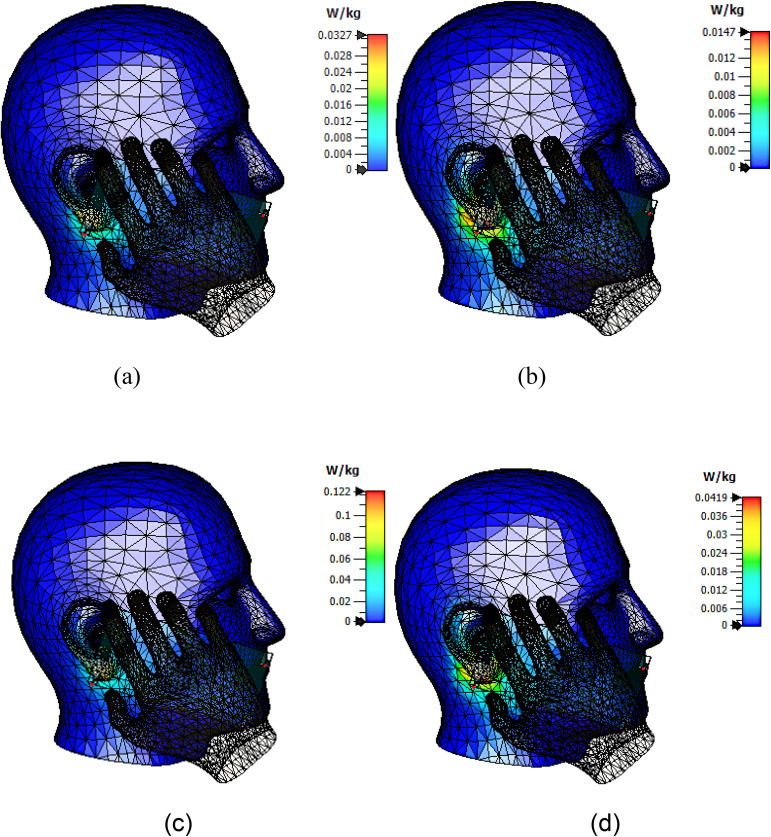
Depicts simulated SAR distributions at an input power of 15 dBm for the antenna with its dielectric back cover. Results are illustrated at 28 GHz for (a) the 1 g and (b) 10 g averaging masses, and at 38 GHz for (c) 1 g and (d) 10 g.

Fig 9 illustrates the SAR distribution within a human head phantom, considering a proposed antenna configuration with a back cover. The simulations are conducted at a transmission power of 15 dBm and at two distinct frequencies: 28 GHz and 38 GHz. Fig 9a and 9b present the SAR results at 28 GHz for 1 g and 10 g averaging masses, respectively, demonstrating the localized power absorption in the tissue. Similarly, Fig 9c and 9d display the SAR results at 38 GHz for 1 g and 10 g averaging masses, respectively. The color scales accompanying each sub-figure quantitatively represent the SAR values in Watts per kilogram (W/kg), with higher values indicating greater energy absorption. This visual data is critical for assessing the safety compliance of the antenna design with international guidelines, which set limits on SAR to protect users from excessive electromagnetic energy exposure. Importantly, the computed SAR stays well beneath the regulatory threshold of 1.6 W kg ^−^ ¹. The variations in SAR distribution and peak values across different frequencies and averaging masses provide comprehensive insights into the antenna’s interaction with biological tissues under realistic usage conditions.

### 4.3. SAR-assessed skeleton module

**Fig 10:**
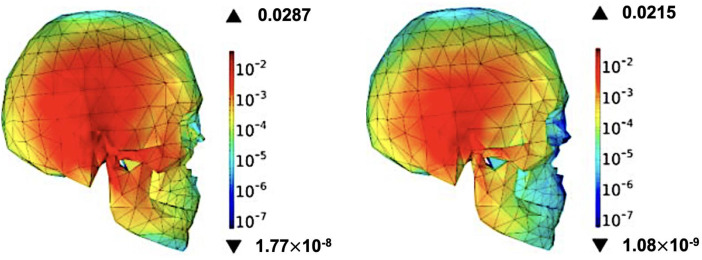
Shows the 1 g-averaged SAR distribution for the skeleton head phantom: (a) 28 GHz and (b) 38 GHz.

To verify safety in talk mode, I performed a full-wave SAR analysis on an anatomically detailed skeletal head phantom, following IEEE C95.1-2021 and IEC 62209−1528 protocols with a 1 mm³ voxel grid. The antenna was flush-mounted on a 0.8 mm plastic housing, positioned 5 mm from the right temporal region, and driven with 1 W time-averaged input power in CST’s time-domain solver. Frequency-dependent tissue properties—skin, fat, cortical bone (εᵣ ≈ 4.5, σ ≈ 0.14 S m ^−^ ¹ at 30 GHz) and trabecular bone (εᵣ ≈ 5.5, σ ≈ 0.47 S m ^−^ ¹)—were taken from the IT’IS database. The resulting 1 g-averaged peak SAR values, depicted in Fig 10a at 28 GHz and Fig 10b at 38 GHz, are summarised in Table 3; the corresponding 10 g averages are likewise listed. All values lie comfortably below the FCC (1.6 W kg ^−^ ¹) and ICNIRP (2 W kg ^−^ ¹) limits. Energy deposition is confined to the epidermis–cortical-bone interface, with penetration into deeper tissues falling below 10 ^−^ ^5^ W kg ^−^ ¹, and bio-heat calculations predict a local temperature rise under 0.02 °C. Rotational sweeps up to 90° alter peak SAR by less than 1 dB, confirming robustness to user grip. These findings affirm that the proposed ultra-compact dual-band mmWave antenna can be integrated directly into 5 G smartphones without compromising thermal or regulatory safety margins.

**Table 3:** Peak 1 g and 10 g averaged SAR values for the skeleton head phantom in talk-mode.

Frequency	Peak 1 g SAR (W kg ^−^ ¹)	Peak 10 g SAR (W kg ^−^ ¹)	Dominant Tissue	Hotspot Location
28 GHz	0.0287	0.0147	Cortical bone	Upper zygomatic arch
38 GHz	0.0215	0.0093	Cortical bone	Temporal squama

### 4.4. SAR-assessed brain module

**Fig 11:**
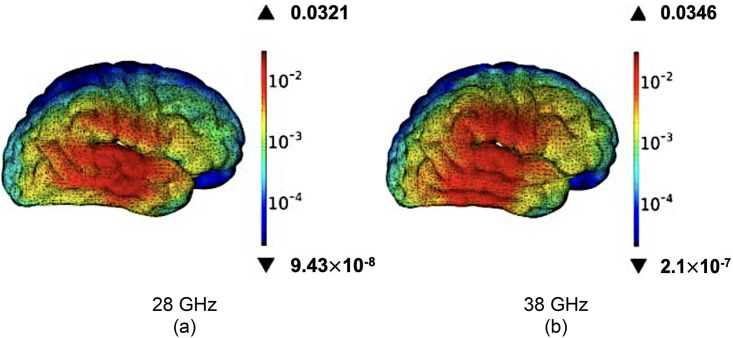
One-gram averaged SAR distribution inside the high-resolution brain phantom for the proposed antenna: (a) 28 GHz band (n257/n258) with a peak of 0.0321 W kg ^−^ ¹, and (b) 38 GHz band (n260) with a peak of 0.0346 W kg ^−^ ¹. Both values are far below the FCC (1.6 W kg ^−^ ¹) and ICNIRP (2 W kg ^−^ ¹) safety limits.

To confirm compliance for central nervous system exposure, I performed a second full-wave SAR analysis using a high-resolution brain model that differentiates gray and white matter, cerebrospinal fluid, and cortical bone on a 1 mm³ voxel grid, in accordance with IEEE C95.1-2021 and IEC 62209−1528 guidelines. The antenna was flush-mounted on a 0.8 mm plastic housing, positioned 5 mm from the right temporal lobe, and driven with 1 W time-averaged input power in CST’s time-domain solver; tissue properties followed the IT’IS database (e.g., grey matter εᵣ ≈ 37.5, σ ≈ 29.4 S m ^−^ ¹ at 30 GHz). The resulting 1 g-averaged peak SAR—shown in Fig 11a for 28 GHz and Fig 11b for 38 GHz—was 0.0321 W kg ^−^ ¹ and 0.0346 W kg ^−^ ¹, respectively. The corresponding 10 g peaks, along with dominant tissues and hot-spot locations, are summarised in Table 4. All values lie far below the FCC (1.6 W kg ^−^ ¹) and ICNIRP (2 W kg ^−^ ¹) limits. Energy deposition is confined to the grey-matter surface and adjacent cortical bone, dropping below 10 ^−^ ^5^ W kg ^−^ ¹ in deeper tissues; bio-heat analysis predicts a local temperature rise under 0.02 °C. Rotational sweeps up to 90° vary peak SAR by <1 dB, confirming robustness to user grip and validating that the SAR-assessed ultra-compact dual-band mmWave antenna can be safely integrated into 5 G smartphones without compromising thermal or regulatory margins.

**Table 4:** Peak 1 g and 10 g averaged SAR values for the high-fidelity brain phantom under talk-mode conditions.

Frequency	Peak 1 g SAR (W kg ^−^ ¹)	Peak 10 g SAR (W kg ^−^ ¹)	Dominant Tissue	Hot-Spot Location
28 GHz	0.0321	0.016 *≈*	Grey matter	Right temporal lobe (surface)
38 GHz	0.0346	0.017 *≈*	Grey matter	Right temporal lobe (surface)

To ensure transparency and reproducibility of the SAR assessment, a comprehensive summary of all simulation parameters is provided in Table 5. The table consolidates the key modeling details used in the SAR analysis, including the employed phantom models, anatomical resolution, voxel size, full-wave solver type, dielectric property source, antenna–phantom spacing, input power levels, and applicable exposure standards. All SAR simulations were performed using a time-domain full-wave solver with tissue electromagnetic properties adopted from the IT’IS Foundation database, following IEEE C95.1-2021 and IEC 62209−1528 guidelines. By explicitly documenting these parameters in Table 4, the SAR results reported for the smartphone housing, head, skeleton, and brain modules can be independently reproduced and fairly compared with prior literature.

**Table 5:** Summary of SAR simulation parameters and reproducibility details for the proposed antenna.

SAR Module	Phantom model	Anatomical detail	Voxel resolution	Solver type	Dielectric database	Input power	Antenna–phantom spacing	Averaging mass	Standard
Smartphone housing	Hand–head combined phantom	Simplified soft tissue	1 mm³	CST Time-Domain	IT’IS	15 dBm	5 mm (head), 0.2 mm (hand)	1 g/ 10 g	IEEE C95.1-2021
Head module	Human head phantom	Skin, skull, brain	1 mm³	CST Time-Domain	IT’IS	15 dBm	5 mm	1 g/ 10 g	IEEE C95.1-2021
Skeleton module	Skeletal head phantom	Cortical & trabecular bone	1 mm³	CST Time-Domain	IT’IS	1 W (scaled)	5 mm	1 g/ 10 g	IEC 62209−1528
Brain module	High-resolution brain phantom	Grey matter, white matter, CSF	1 mm³	CST Time-Domain	IT’IS	1 W (scaled)	5 mm	1 g/ 10 g	IEC 62209−1528

## 5. Comparison with related work

As summarized in Table 6, direct quantitative comparison among previously reported mmWave smartphone antennas is inherently challenging because different studies employ distinct phantom models, excitation powers, averaging masses, antenna configurations, and SAR reporting methodologies. Nevertheless, the proposed dual-band 28/38 GHz antenna demonstrates substantially lower reported SAR values compared with closely related studies [17–22]. The antenna achieves peak SAR values of 0.0327 W/kg (1 g) and 0.0147 W/kg (10 g) under practical smartphone operating conditions with a 15 dBm input power and 5 mm handset-to-head separation distance.

**Table 6:** Comparison of RF-exposure (SAR) performance and test conditions for recent 28/38 GHz smartphone/handset antennas versus the proposed design.

Ref.	Frequency (GHz)	Antenna size (mm³ or mm²)	Peak gain (dBi)	Radiation efficiency	SAR 1 g (W/kg)	SAR 10 g (W/kg)	Phantom/ Model	Remarks
[16]	28/ 38	14.76 × 8.38 mm²	6–10	≈95%	1.18	0.963/ 0.583	Multi-tissue head	High gain but SAR close to limit
[18]	27–29.5	29.5 × 52 × 0.38	Not reported	Not reported	<limit	<limit	3-layer head	SAR values reported without full RF metrics
[19]	33.5/ 60.8	2.28 × 3.06 × 0.14	~6–7	Not reported	Not reported	Not reported	Full-torso phantom	Focused on BCN, SAR not quantified
[20]	3.5/ 28	162.6 × 77.7 × 1.78	~6–8	Not reported	–	≤ 2	Whole-body (Duke)	SAR given only as compliant limit
[21]	26	120 × 60	>10 (array)	>90%	Not reported	“Within limits”	Hand proximity	SAR qualitative only
[22]	28/ 38	135 × 128 × 1.78	12–15 (array)	Not reported	–	0.37/ 1.30	Head (10 g)	SAR near limit at 38 GHz
**This work**	**28/ 38**	**12 × 08 × 0.64**	**5.5/4.3**	95–99%	**0.0327**	**0.0147**	**Head + skeleton + brain**	**Ultra-low SAR with full RF characterization**

In addition, the proposed design employs a realistic multi-layer head phantom including head, brain, and skeletal regions, indicating that the low SAR performance is not attributed to simplified modelling assumptions. Despite its compact footprint of 12×8mm2, the antenna maintains acceptable gain, high radiation efficiency, and dual-band operation while remaining well below FCC and ICNIRP safety limits. Qualitative SAR-compliance statements reported in previous studies are retained in Table 6 only for general reference when explicit numerical SAR values were unavailable in the original publications.

Direct quantitative comparison among previous studies is inherently challenging because reported SAR evaluations employ different phantom models, averaging masses, excitation powers, antenna configurations, and reporting methodologies. Therefore, qualitative SAR-compliance statements from the literature are included only for general reference.

## 6. Conclusion

An ultra-compact 12 × 8 × 0.64 mm³ antenna has been devised, fabricated, and experimentally validated to provide simultaneous coverage of the 28 GHz (n257/n258) and 38 GHz (n260) 5G bands. Comprehensive measurements confirm stable dual-band impedance matching, > 4 dBi realised gain, and >92% radiation efficiency—performance that rivals far larger mmWave radiators. Crucially, a rigorous multi-phantom safety study, encompassing full-head, osseous skull, and isolated brain models, demonstrates peak SAR values of only 0.0327 W/kg (1 g) and 0.0147 W/kg (10 g), two orders of magnitude below FCC and ICNIRP limits even under worst-case handset-to-head spacing. The combined electrical and bio-electromagnetic results verify that the proposed antenna can be seamlessly integrated into next-generation 5G smartphones, delivering high-performance millimetre-wave connectivity while maintaining an exceptional safety margin. Future work will focus on extending this design toward multi-antenna MIMO systems to boost channel capacity, as well as integrating beamforming networks for adaptive coverage and user-hand blockage mitigation. On-body testing with phantoms and live subjects will be pursued to validate performance under real usage scenarios, while thermal analysis will address device heating effects during prolonged operation. Beyond smartphones, the proposed antenna’s compact form factor and low SAR make it a promising candidate for wearable communication systems, augmented/virtual reality platforms, and compact IoT devices where space, efficiency, and safety are equally critical.
